# Bimodal Imaging Probes for Combined PET and OI: Recent Developments and Future Directions for Hybrid Agent Development

**DOI:** 10.1155/2014/153741

**Published:** 2014-04-16

**Authors:** Uwe Seibold, Björn Wängler, Ralf Schirrmacher, Carmen Wängler

**Affiliations:** ^1^Biomedical Chemistry, Department of Clinical Radiology and Nuclear Medicine, Medical Faculty Mannheim of Heidelberg University, Theodor-Kutzer-Ufer 1-3, 68167 Mannheim, Germany; ^2^Molecular Imaging and Radiochemistry, Department of Clinical Radiology and Nuclear Medicine, Medical Faculty Mannheim of Heidelberg University, 68167 Mannheim, Germany; ^3^McConnell Brain Imaging Centre, Montreal Neurological Institute, McGill University, Montreal, QC, Canada H3A 2B4

## Abstract

Molecular imaging—and especially positron emission tomography (PET)—has gained increasing importance for diagnosis of various diseases and thus experiences an increasing dissemination. Therefore, there is also a growing demand for highly affine PET tracers specifically accumulating and visualizing target structures in the human body. Beyond the development of agents suitable for PET alone, recent tendencies aim at the synthesis of bimodal imaging probes applicable in PET as well as optical imaging (OI), as this combination of modalities can provide clinical advantages. PET, due to the high tissue penetration of the *γ*-radiation emitted by PET nuclides, allows a quantitative imaging able to identify and visualize tumors and metastases in the whole body. OI on the contrary visualizes photons exhibiting only a limited tissue penetration but enables the identification of tumor margins and infected lymph nodes during surgery without bearing a radiation burden for the surgeon. Thus, there is an emerging interest in bimodal agents for PET and OI in order to exploit the potential of both imaging techniques for the imaging and treatment of tumor diseases. This short review summarizes the available hybrid probes developed for dual PET and OI and discusses future directions for hybrid agent development.

## 1. Introduction

Within the last decades, the development of new radiotracers for PET imaging has experienced an enormous progress due to its enormous specificity and sensitivity in the visualization of target tissues. Thus, a rising number of valuable compounds applicable in cardiologic, neurologic, and especially oncologic imaging were developed. However, PET alone displays a limited spatial resolution of 1–3 mm in clinical practice and also is not able to allow a morphological correlation of the tracer accumulation which is however especially crucial in case of tumor diagnosis, localization, and staging. Thus, almost all clinical PET systems sold within the last years are combinations of PET and computed tomography (CT) systems, integrating the strengths of both modalities: the high specificity and sensitivity of PET making already functional changes in tissues visible at a very early stage of disease and the detailed morphologic information provided by CT [1]. Most recently, also combined clinical PET/MRI (magnetic resonance imaging) systems are commercially available. The MRI modality provides an even higher resolution and soft tissue contrast than CT, allowing for a functional imaging without causing any additional radiation burden to the patient. In combination with the very high sensitivity and specificity of PET, an almost ideal combined imaging modality is obtained for the whole-body imaging of patients [2] although the number of hybrid agents applicable in PET/MR imaging is very limited so far.

Despite these favorable properties of PET/CT and also PET/MRI systems in whole-body imaging for the identification of target structures, these modalities exhibit certain limitations: after having specifically identified and localized a tumor target tissue, the resection of the tumor mass is difficult due to the intricate intraoperative identification of tumor margins and small metastases. Additionally, the identification of the sentinel lymph node (SLN) which is often resected for histology is not trivial. For this purpose, another combination of imaging modalities could be of special interest, namely, the combination of PET with optical imaging (OI).

**Figure 1:**
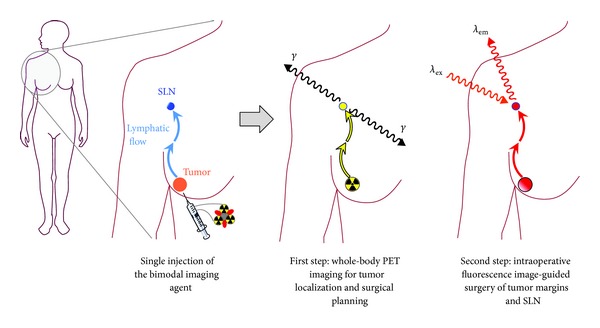
Schematic depiction of the operation principle of a PET/OI hybrid compound. After being applied to the patient in a single injection, an initial whole-body PET scan is performed, identifying and localizing tumor and potential metastases, thus serving as a tool for surgery planning. During the following surgical intervention, the same compound—having accumulated in the target tumor areas over time—can be used as a marker for intraoperative image-guided surgery of the respective malignant tissues.

Although OI is a modality with restricted applicability for whole-body* in vivo* imaging due to the limited tissue penetration of the light emitted by the fluorescent probe, it is a valuable methodology for surface imaging applications such as intraoperative image-guided surgery due to its favorable spatial resolution and sensitivity [3–5]. Thus, a combined bimodal imaging consisting of an initial PET scan using *γ*-radiation with a high tissue penetration range to identify and localize tumor lesions throughout the body and a subsequent intraoperative OI in order to identify tumor margins and infected lymph nodes can result in a significant clinical improvement [6–8]. Especially in breast and prostate carcinomas as well as melanomas, the prognosis strongly depends on the presence of lymph node metastases [9–11]. However, the secure intraoperative identification of sentinel and infected lymph nodes is crucial for efficient diagnosis and treatment but is difficult if the surgeon can only rely on abnormal visual appearance and palpation to discriminate between lymph nodes and surrounding tissues or to identify infected nodes. The use of a specific tumor-accumulating agent which can be visualized during surgery by optical imaging techniques emitting light which can penetrate tissue in a reasonable range (so that a target node can be detected even if not already fully surgically exposed) would mean a significant improvement for surgery (Figure 1).

The development of new combined imaging techniques however also requires the development of the respective hybrid imaging agents that are suitable for all involved imaging modalities. Thus, considerable research has been conducted in this field of hybrid contrast agents over the last years [3, 4, 12–15].

For combined PET and optical imaging, in principle, the use of two separate molecular markers, one for PET and one for OI (instead of using a hybrid imaging agent), would also be possible. However, this is no optimal approach as both agents are likely to exhibit differing biodistribution and pharmacokinetic properties (especially in cases of relatively small, specifically accumulating biomolecules such as peptides). Hence, to achieve reliable results that are comparable between both imaging modalities, a hybrid marker has to be applied.

It has to be kept in mind that optical imaging is not fully quantifiable as it is surface-weighted due to absorbance and scattering of the photons by tissue penetration (especially when imaging deep tissues exhibiting a low imaging agent accumulation) and thus cannot be fully correlated to PET imaging data [16–19]. As PET, however, is fully quantifiable and used for whole-body imaging whereas OI is used for intraoperative imaging purposes only in order to identify tumor tissues (tumor margins, small metastases, and infected lymph nodes), the quantification of optical signals is no critical criterion.

For OI, different classes of reporter probes detectable by optical imaging techniques can in principle be used in a hybrid PET/OI agent: (i) fluorescent proteins that can be detected by bioluminescence imaging (BLI), (ii) *γ*-emitting radionuclides that can be visualized by Cherenkov luminescence imaging (CLI: luminescence that can be observed when a particle travels faster than light in the examined medium), (iii) fluorescent small dye molecules that can ideally emit near infrared light, and (iv) quantum dots which are semiconductor nanocrystals consisting of Cd/Te or Cd/Se materials and whose emission characteristics can be tailored by particle size. For all these probes that can be used in the development of a hybrid PET/OI agent, substances emitting light of the near-infrared and infrared spectrum (700–900 nm) are most useful, as light of these wavelengths exhibits the highest tissue permeability of several mm to cm* in vivo* [20, 21].

Large proteins such as GFP (green fluorescent protein) or RFP (red fluorescent protein) are in principle applicable in the synthesis of a hybrid compound. However, they are structurally demanding and would most possibly have a severe impact on the pharmacokinetic properties of the resulting imaging agent. Thus, it is only conceivable to use these compounds in combination with particle carriers. Furthermore, the quantum yield of these proteins is rather limited and they do not enable near-infrared photon emissions [22], further restricting the use of fluorescent proteins in hybrid optical imaging agents.

In contrast, CLI using different positron-emitting radionuclides has been proposed as a favorable optical imaging technique for imaging-guided surgery [23]. This technique does not require the conjugation of an additional fluorescent compound in order to obtain a bimodal imaging agent. This is favorable as an additionally conjugated fluorescent dye can—if susceptible to the radiolabeling conditions applied—interfere with the radiosynthesis or result in a significant alteration of the pharmacokinetic properties of the resulting hybrid compound. Unfortunately, using the Cherenkov luminescence imaging approach, one of the most valuable properties of combined PET/OI probes to be applied in intraoperative imaging, namely, the consecutive detection via PET and the subsequent later resection of the tumor, cannot be utilized. Using a hybrid compound consisting of a fluorescent dye in addition to a radionuclide, the optical intraoperative imaging can be performed delayed in time after identifying and localizing the tumorous tissue by a whole-body PET scan. By this procedure, the radionuclide at least partially decayed before surgery, resulting in no or only low radiation burden to the surgeon during intraoperative imaging and resection. In contrast, using CLI for intraoperative imaging can result in a significant radiation burden as is indicated by a recent study, systematically investigating the potential of CLI in a preclinical setting. In this work—when imaging an ^124^I activity depot located subcutaneously in 4 mm depth—an activity concentration of at least 0.3 mCi/mL (11.1 MBq/mL) was necessary to obtain a detectable signal [24].

In most of the reported bimodal hybrid compounds for PET/OI, small fluorescent dyes or quantum dots are thus applied as they produce no ionizing radiation and are relatively stable under physiological conditions [25–27]. This allows for an image-guided surgery even after the decay of the radionuclide. In addition, small fluorescent dye molecules exhibit the advantage of being relatively small in size and thus result in a less prominent influence on the binding parameters of the carrier molecule which is especially important for the derivatization of small and medium-sized biomolecules.

## 2. Examples of Dually Labeled Agents Applicable in Hybrid* In Vivo* PET and Optical Imaging

Besides hybrid agents for combined PET/OI, also markers for dual SPECT/OI have been developed over the last years, comprising dually labeled antibodies [28, 29], peptides [30–35], a nontargeted small molecule [36], and nanoparticles [37–40]. However, as PET is—in contrast to SPECT—fully quantifiable and exhibits a much higher sensitivity than the latter, the main focus in this young field of bimodal probe development for use in nuclear medicine and optical imaging lies on the development of PET/OI agents, having a greater potential for a possible clinical application.

### 2.1. Nontargeted Small Molecules

**Figure 2:**
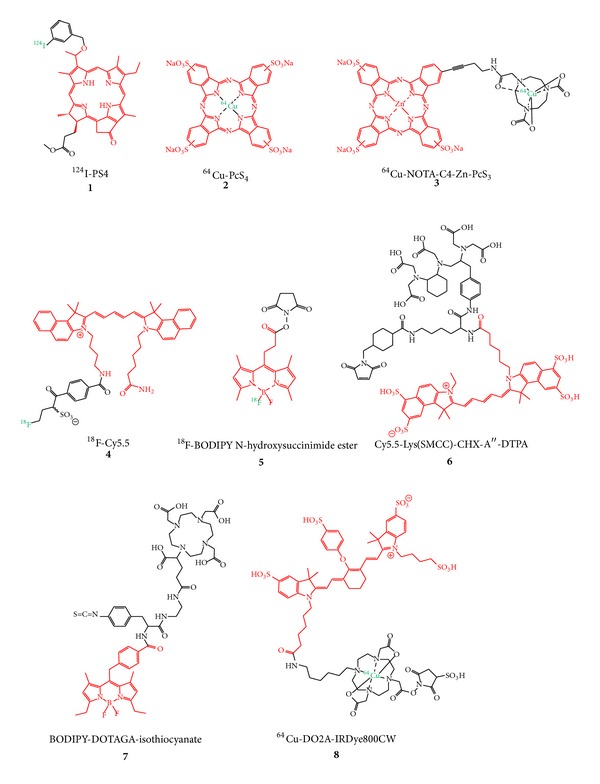
Structures of small molecule-based bimodal labels developed for hybrid imaging with PET and OI (fluorescent dyes are depicted in red and PET nuclides in green).

Apart from targeted and nontargeted probes based on different biomolecule or nanoparticle carriers developed for a mostly tumor target-specific accumulation, the synthesis of several small molecule-based bimodal labels was reported. These are intended to be used directly without any further targeting for imaging (Figure 2,** 1**–**3**) or could serve as a basis for a future bimodal labeling of biologically active compounds such as antibodies and other proteins (Figure 2,** 5**–**8**).

The imaging agents** 1–3** [41–44] depicted in Figure 2 are based on porphyrin or phthalocyanine derivatives which can show a significant accumulation in tumor tissues and can be used as photosensitizers thus being applicable in photodynamic therapy. These porphyrin and phthalocyanine derivatives were radiolabeled with ^124^I and ^64^Cu in different positions, respectively, and subjected to tumor xenograft mice for* in vivo* evaluation of their PET and/or optical imaging characteristics. Due to the missing tumor targeting entity, the observed tumor accumulations were faint and also a high unspecific accumulation of the compounds in nontarget organs such as liver, spleen, gut, lung, and blood was observed [41, 42, 44], limiting the usefulness of these compounds for* in vivo* tumor imaging. The ^18^F-labeled Cy5.5 derivative** 4** was synthesized in a proof of concept approach to demonstrate the applicability of a new secondary ^18^F-labeling precursor for the radiolabeling of even sensitive molecules such as cyanine dyes and was thus not investigated regarding its* in vivo* characteristics. It could however be useful as hybrid label if functionalized for a bioconjugation and introduced into a targeting vector [45].

In contrast to compounds** 1**–**4**, hybrid agents** 5**–**8** [46–49] are not intended to be used directly for an* in vivo* application but for conjugation to a specifically accumulating agent such as a peptide, antibody, or antibody fragment by different reactive functional groups (active esters, maleimide, and isothiocyanate). By this approach, the concomitant introduction of the radio- and the fluorescent label into the biomolecule is enabled and the resulting hybrid probe can be used in a targeted* in vivo* imaging application. However, the application of such a hybrid label for the derivatization of a specifically accumulating carrier molecule as well as the subsequent radiolabeling and* in vivo* evaluation of the so obtained dually labeled imaging probe was so far only shown for** 8**. For this purpose, an anti-EpCAM antibody was first reacted with DO2A-IRDye800CW-sulfo NHS ester, radiolabeled with ^64^Cu (**8**), and finally evaluated in a proof of concept study in PC-3 xenograft mice [49]. Unfortunately, only near-infrared fluorescence imaging (NIR-FI) whole-body* in vivo* images and no PET data were shown in this study which is most probably attributable to the foreseeable insufficient stability of the ^64^Cu-DO2A complex [50], resulting in very high liver and blood accumulations of the radionuclide compromising the* in vivo* PET data. In addition, also the NIR-FI data point to a predominant liver and also high kidney as well as lung accumulation at 40 h p.i. of the dual-labeled antibody, limiting its potential for hybrid* in vivo* tumor imaging. Thus, though not directly applicable* in vivo*, these small molecule-based hybrid imaging probes reflect the high interest in this relatively new field of hybrid PET/OI agent development.

### 2.2. Dually Labeled Small Molecules and Peptides Intended for Target-Specific Accumulation and Bimodal Target Visualization by PET/OI

The introduction of a fluorescent dye together with a radiolabel (which is either covalently attached or complexed by a chelator system) can result in a significant structural alteration especially in case of rather small target-specific molecules. Nevertheless, attempts have been made to synthesize such dually labeled small molecules and peptides, as they usually display fast pharmacokinetics and target accumulations and a rapid clearance from nontarget tissues, in principle resulting in favorable high-contrast images.

**Figure 3:**
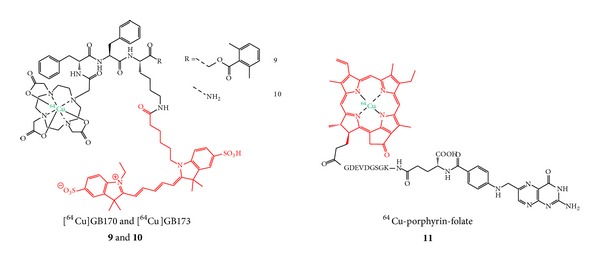
Structures of hybrid small molecule agents intended for specific accumulation in tumor tissues.

Especially for the studied small molecules (Figure 3,** 9**–**11**), the structural change by introducing two labels was shown to result in high background and low specific tumor accumulations. The main excretory organ was in all cases the liver, for which a very pronounced uptake of the radiolabeled substances was observed, but also kidneys, spleen, and intestines showed high accumulations of the tracers, hampering a high tumor uptake and thus efficient tumor visualization with PET [51, 52].

Interestingly, it could be shown in the study dealing with the dually labeled cysteine cathepsin substrates [^64^Cu]**9** and [^64^Cu]**10** that the introduction of the NIR dye can result in a favorable prolonged circulation compared to the ^64^Cu-DOTA-modified analog not comprising a fluorescent dye [52]. This positive effect of fluorescent dye conjugation could be confirmed by another study [16]. This prolonged circulation was described to result not only in a higher unspecific accumulation in all organs but also in a significantly higher and at least in part specific tumor accumulation [52] presumably due to a higher interaction probability of the hybrid agent with its target.

Despite the disappointing results obtained with PET imaging of the ^64^Cu-labeled porphyrin-folate conjugate** 11**, a clear tumor visualization was possible with fluorescence imaging (FI) after 24 h p.i. which can be attributed to the fact that the tumors were rather large and located directly under the skin. Furthermore, the tumors were—from all tissues—located nearest to the detector system, minimizing the absorbance and scattering of the fluorescent light emitted from the tumors whereas the photons emitted from the excretory organs were most probably strongly attenuated [51].

**Figure 4:**
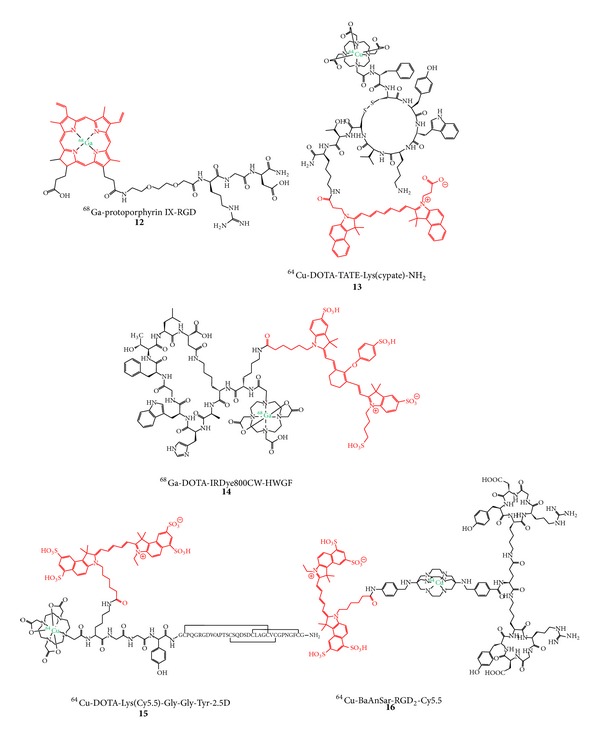
Structures of dually labeled PET radionuclide and fluorescent dye comprising peptides developed for* in vivo* tumor imaging with PET and OI.

Due to their larger molecular size, peptides are in principle more likely to tolerate a derivatization with two different labels in terms of receptor binding and* in vivo* pharmacokinetics. This theoretical tendency seems in fact to be reflected in the results obtained for dually labeled peptides. From the dually labeled PET radionuclide and fluorescent dye comprising peptides available for tumor imaging so far (**12**–**16**, Figure 4), 4 compounds, namely,** 13–16**, were evaluated* in vivo* regarding their biodistribution properties and tumor visualization abilities [16, 17, 25, 26, 53].

The results obtained in these studies seem to point to a more favorable biodistribution together with higher tumor to background ratios and higher tumor targeting specificity in case of larger dually labeled peptidic targeting vectors. It could, for example, be shown that for Tyr^3^-octreotate (TATE), derivatized at the N-terminus with ^64^Cu-DOTA and at the C-terminus with a NIR dye (**13**), on the one hand encouraging* in vitro* binding results to A427-7 tumor cell membranes with *K*_*i*_ values of 0.43 nM for TATE and 11.5 nM for ^nat^Cu-**13 **could be obtained but that the agent was on the other hand not able to visualize the respective A427-7 tumor* in vivo* in xenograft mice at 24 h p.i. with FI [26]. Moreover, the low tumor uptake (only reaching a tumor-to-blood ratio of about 2) could not be blocked by cold peptide in biodistribution studies with ^64^Cu-**13**, pointing to an unspecific tumor accumulation caused by the EPR effect (enhanced permeability and retention effect: a passive tumor-targeting process that results in an unspecific uptake of compounds due to a more permeable tumor vasculature and efficient diffusion through the tumor interstitium). Overall, a significant accumulation of this compound could only be observed in liver (16.824 ± 1.520%ID/g), spleen (8.069 ± 1.808%ID/g), and lung (1.428 ± 0.738%ID/g) after 1 h p.i. (for comparison: tumor accumulation was 0.287 ± 0.046%ID/g), pointing to a too pronounced overall lipophilicity of the compound for a successful* in vivo* application.

Similar effects were shown for** 14**, which was developed to visualize MMP2 and 9* in vivo* [25]. However, in a heterotopic ossification model activating MMP9, a target visualization could not be achieved by PET imaging. Using NIR-FI, the ossification site could be visualized, but no whole-body images were shown limiting the informative value of these images.

In contrast to these latter studies, two examples of very promising peptidic hybrid compounds for PET and OI were reported. One of these—consisting of an *α*_*ν*_ *β*_3_ and *α*_*ν*_ *β*_5_-affine knottin peptide targeting tumor angiogenesis, derivatized with Cy5.5 and DOTA via an amino acid spacer—was radiolabeled with ^64^Cu (**15**) and successfully used for specific* in vivo* PET and NIR-FI of an integrin-positive U87MG tumor in xenograft mice [17]. These favorable results were achieved although the* in vitro* binding data indicated an adverse influence of the derivatization of the peptide with NIR dye and chelator compared to a monolabeling with DOTA or NIR dye alone. Interestingly, comparing the ^64^Cu-DOTA-monolabeled knottin peptide with the dual-labeled one regarding* in vivo* biodistribution with PET, both compounds achieve tumor-to-background ratios (TBR) of ∼4.5. However, these comparable ratios were found at different time points: the ^64^Cu-DOTA-monolabeled peptide reaches this TBR already at 4 h p.i. whereas the same TBR is achieved by the dually labeled peptide** 15** at 24 h p.i., indicating—as described before—a retention-prolonging effect of the conjugated NIR dye. This is confirmed by the corresponding NIR-FI experiment comparing the NIR-monolabeled peptide with the dually-labeled one** 15** which both reach the TBR of ∼4.5 at 24 h p.i.

A very encouraging example of a dually labeled hybrid compound was described recently, consisting of a cRGD-dimer (serving as tumor-targeting vector) and Cy5.5 which is connected to the peptidic part via a sarcophagine-derived chelator used for ^64^Cu-labeling [16]. The radiolabeled compound ^64^Cu-**16 **was successfully used for the* in vivo* imaging of integrin-rich U87MG tumors in a xenograft mouse model, showing a high tumor uptake together with a stable tumor retention (6.41 ± 0.28, 6.51 ± 1.45, and 5.92 ± 1.57%ID/g at 1, 4, and 20 h* p.i.*, resp.), resulting in the highest tumor-to-background ratios of ~7 at 20 h p.i. As described before, this NIR dye-labeled compound ^64^Cu-**16** showed a prolonged circulation together with a higher tumor accumulation compared to the corresponding, nonfluorescent-labeled derivative [16]. Furthermore, ^64^Cu-**16** was used for image-guided resection of the tumor in the same animal model and showed—in contrast to the PET images displaying a homogeneous tumor areal due to the physically limited spatial resolution of ^64^Cu—the presence of a metastasis near the primary tumor, impressively demonstrating the advantages of intraoperative optical imaging and the synergistic effects of PET combined with OI.

These favorable* in vivo* imaging results found for** 15** and** 16** are probably a result of two different effects: the large size of the peptidic targeting vector relative to both labels and also the introduction of both labels in only one position of the peptidic moiety, limiting their influence on the overall biodistribution compared to two labels introduced in different positions of the peptide. Thus, due to the strong potential influence of two labeling moieties introduced, the ligand design has to be carefully considered especially when derivatizing peptides.

### 2.3. Fluorescent and Radiolabeled Antibodies for Combined PET/OI

**Figure 5:**
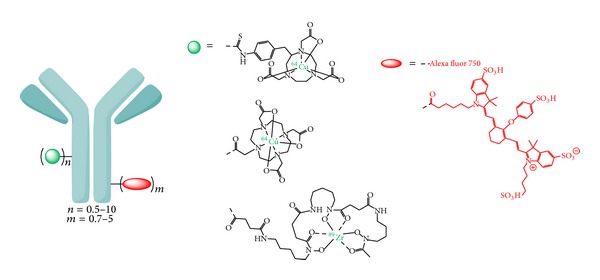
Schematic depiction of dually labeled antibodies developed for* in vivo* hybrid PET/OI of tumors.

Antibodies with their slow pharmacokinetics and very high target specificity should be well suited as targeting vectors for a dual-labeling approach with a PET nuclide and a NIR dye as they exhibit a more complex structure than small molecules and peptides resulting in a less strong alteration of structure, binding characteristics, and thus biodistribution properties by the concomitant conjugation of two labels. Several different antibodies have been derivatized with desferrioxamine [54–56] for ^89^Zr-labeling, NOTA [57] or DOTA [18, 58, 59] for ^64^Cu-labeling, and the NIR dyes 800CW [54–59] or Alexa Fluor 750 [18] (Figure 5).

In all studies, it could be demonstrated that the number of introduced derivatization sites has a crucial effect on the biodistribution characteristics of the obtained hybrid compounds. One study, for example, describes the derivatization of an anti-CD20 IgG with ~10 chelators and ~2 fluorescent dyes, resulting in an unfavorable biodistribution of the hybrid compound in lymphoma-bearing mice, showing a very high liver and spleen accumulation of the antibody. Consequently, only a moderate tumor uptake was observed resulting in only poor tumor visualization* in vivo* [18]. Reducing the number of introduced labels, radionuclide chelator and fluorescent dye, to ~2 per trastuzumab molecule, improved results could be obtained in 4T1.2neu/R tumor-bearing xenograft mice, allowing for a tumor visualization with PET as well as NIR-FI at 24 h p.i., although tumor-to-muscle ratios of only ~2.5 were obtained [58]. Reducing the number of both labeling moieties to 1 per anti-CD105 antibody molecule, the tumor-to-muscle ratios could be improved to ∼7 in 4T1 tumor xenograft mouse models [56]. However, besides a tumor uptake of ∼10%ID/g, high liver, spleen, and blood uptakes of ∼16%ID/g, ∼8%ID/g, and ∼11%ID/g were observed at 48 h p.i., respectively, impairing the* in vivo* imaging results. Nevertheless, the observed tumor uptakes were no result of the EPR effect alone but also of a specific binding, as they could be blocked by about 50% by coapplication of unlabeled antibody.

Other studies, limiting the number of introduced labels to a minimum of 0.5–0.9 equivalents of each labeling moiety per antibody, found even more favorable biodistribution properties such as a slowed clearance, lower liver, and higher and prolonged tumor uptakes resulting in a clear visualization of the tumor mass* in vivo* at 48 h p.i. with PET as well as NIR-FI [54, 57]. Besides the observed impaired biodistribution properties of high liver and spleen uptake* in vivo* when conjugating several fluorescent dye molecules per antibody, a conjugation of several dyes also results in a fluorescence quenching effect and thus a decreased overall fluorescence intensity, being detrimental to a successful* in vivo* NIR-FI of the target tissue [54, 57]. These studies furthermore investigated the correlation between organ uptakes determined by* in vivo* PET and* in vivo* or* ex vivo* NIR-FI. The PET data in these studies served as reference parameters as PET is fully quantifiable. It was found that the deviations in measured organ uptakes were higher for the NIR-FI data obtained* in vivo* than obtained* ex vivo*. Also, the deviations were higher for deeper tissues, pointing to a significant scattering and absorbance of the NIR light, limiting the quantification of tissue uptakes by fluorescence imaging and necessitating the quantification of organ uptakes by PET. This is, however, no limitation for optical imaging in terms of intraoperative imaging settings where only qualitative images are required for a successful tumor resection.

Besides the described general findings regarding the negative influence of a high number of conjugated labeling moieties on the biodistribution properties of derivatized antibodies, one study systematically investigated the influence of the number of conjugation sites on the biodistribution of dual-labeled antibodies [55]. There, the EGFR and VEGF targeting antibodies cetuximab and bevacizumab were initially derivatized with on average 0.5 desferrioxamine chelators, followed by an introduction of 0.5 to 5 800CW NIR dyes, and the biodistribution of the resulting hybrid imaging compounds was determined* in vivo* after ^89^Zr-radiolabeling. It could be shown that the antibody uptake into the liver proportionally increased with the number of conjugated dyes, whereas the tumor accumulation decreased to the same extent.

Thus, in order to achieve optimal imaging results using a dually labeled hybrid antibody for PET and NIR fluorescence imaging, the development of a small molecule serving as a dual-label would be of advantage. This molecule could consist of a chelator for radiometal labeling, the NIR dye, and a functionality enabling a concomitant conjugation of both labels in only one position of the antibody, thus limiting its structural change to an absolute minimum.

### 2.4. Nanoparticles as a Platform for Hybrid PET/OI Agents

Nanoparticles—in contrast to biomolecules—exhibit the advantage of possessing a large surface which can easily be modified with functional groups for the conjugation of targeting vectors, radiolabels, and fluorescent dyes. On the other hand, they also face several problems: (i) they necessitate a stable coating for functionalization, (ii) exhibit a long tissue retention, (iii) only insufficient knowledge is available about their toxicity (especially in case of quantum dots, consisting of Cd ions and other potentially toxic metals), metabolism, and excretion, and (iv) they strongly accumulate in the reticuloendothelial system (RES) and thus in liver, spleen, bone marrow, and lymph nodes. Furthermore, the stoichiometry of the conjugated moieties is difficult to control or quantify after reaction. Nevertheless, most of the hybrid compounds developed for dual PET and OI so far are based on nanoparticles as carriers.

The group of nanoparticles applicable as structural basis for hybrid PET/OI agents consists of several different subgroups: polymer-based nanoparticles [60], lipid-based particles such as micelles [61] and liposomes [62], carbon-based systems such as nanotubes [63], and also metal-based nanoparticles such as iron oxide [5, 27, 64–67], silica [8], and upconversion nanoparticles [68, 69] as well as quantum dots (QDs) [70–73].

**Figure 6:**
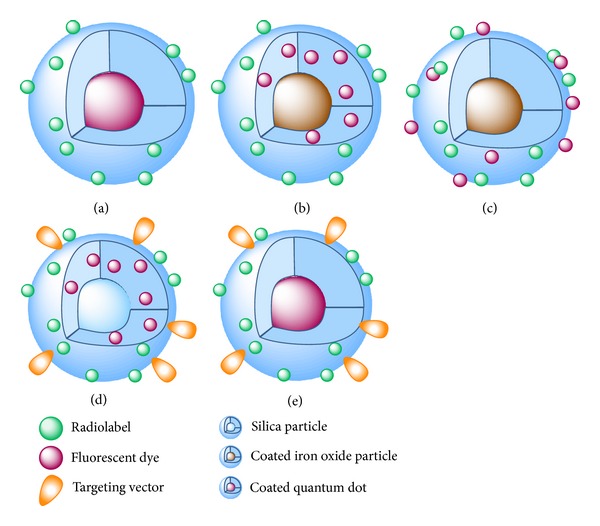
Schematic representation of different kinds of labeled nanoparticles that were already used as hybrid compounds for PET and OI: (a) coated quantum dot loaded with chelating agent for radiometal introduction, (b) coated iron oxide particle with encapsulated fluorescent dye and derivatized on its surface with a chelator for radiolabeling, (c) coated iron oxide particle derivatized on the surface with fluorescent dye and chelator for radiometal labeling, (d) silica nanoparticles with encapsulated fluorescent dye and surficial derivatization with radionuclide and targeting vector, and (e) coated quantum dot loaded with chelating agent for radiometal introduction and a targeting vector.

QDs are fluorescent semiconductor nanocrystals whose fluorescent properties can be influenced by the particle size and composition. Furthermore, they exhibit high quantum yields and photostability [74], making them interesting fluorophores for the development of compounds for hybrid PET/optical imaging when stably radiolabeled with a positron-emitter (Figure 6(a)). Superparamagnetic iron oxide nanoparticles on the other hand are detectable by MRI, enabling a triple-modality imaging with PET/OI and MRI when derivatized with fluorescent dyes and radionuclides (Figures 6(b) and 6(c)) [27, 64]. QDs as well as iron oxide nanoparticles have to be coated with biocompatible materials to render them amenable for an* in vivo* application. This coating can consist of different materials such as SiO_2_ or other inorganic material, dextran, micelles, or polyethylene glycols (PEGs) and furthermore enables a chemical modification of the surface of the particles with dyes, radiolabels, and targeting vectors allowing for a target-specific accumulation (Figures 6(d) and 6(e)). An alternative to the approach of chemical modification of the coating of a nanoparticle with NIR dyes in order to obtain a fluorescent agent is the encapsulation of the fluorophore within the particle coating (Figure 6(b)) which has been shown to result in a much higher fluorescence signal and photostability of the fluorescent dye than a surficial dye conjugation (Figure 6(c)) [8, 27].

An important factor in the design of particles intended for* in vivo* imaging purposes is their sufficient stability over the duration of the examination. Thus, also the radiolabel has to be stably introduced by covalent conjugation (in case of nonmetallic isotopes such as ^18^F or iodine isotopes) or stable complex formation (in case of radiometal ions). As the development of hybrid agents for combined PET and OI is still in the beginning, nanoparticles which do not exhibit a stable radionuclide introduction have also been reported. In these cases, the particles were only incubated with the radionuclide, “trapping” the respective radioisotope by proteins used for coating of the particle surface [61], functional groups such as primary amines [64], ionic interactions for ^18^F-labeling [68, 69], or the use an suboptimal chelator for the applied radiometal [65]. In these cases, liberation of the radionuclide was inevitable, resulting in the expectable unfavorable biodistribution characteristics of the radiolabel and thus low image quality. In other cases, the potential of the labeled nanoparticles was not demonstrated as the agents were applied via intratumoral injection [62] or incubated with tumor cells that in the following could be visualized in animals directly after implantation of the labeled cells [66].

In contrast, also well-designed hybrid nanoparticle probes were described, showing highly promising results and giving directions for further developments.

As already mentioned, the particle coating allows not only for the conjugation of fluorescent dyes and radiolabels but also for modification with a targeting vector such as peptides or proteins for enabling a tumor-specific accumulation and imaging, but only few examples of such targeted particles can be found. Two of them describe the surface-modification of ^64^Cu-labeled QDs with VEGF and c(RGDyK) for* in vivo* imaging of angiogenesis and, in both cases, a VEGFR_2_ and *α*_*v*_ *β*_3_ receptor-specific binding could be demonstrated* in vitro* and* in vivo* [70, 71]. Although the major fraction of the particles was shown to rapidly accumulate in the reticuloendothelial system (which is attributed to their size of about 20 nm), both particles allow a visualization of the tumor entity in the respective tumor xenograft mouse models. Furthermore, they show a targeting-vector dependent accumulation as the respective particles without a VEGF or c(RGDyK) derivatization show only a background level tumor accumulation. Another even more favorable example of a hybrid nanoparticle was described very recently. Small silica particles of 6-7 nm in diameter comprising encapsulated NIR dye Cy5.5 were PEG-coated in order to achieve a higher biocompatibility and lower liver accumulation. These particles were further derivatized on their surface with c(RGDyK) and radiolabeled with ^124^I on the tyrosine moiety of the peptide. These particles were successfully used for whole-body PET imaging for tumor and multiple metastases visualization (showing a very favorable biodistribution without accumulation the RES) as well as intraoperative imaging guidance in a spontaneous melanoma miniswine model [8]. The intraoperative imaging was performed using a hand-held fluorescence imaging camera allowing for real-time fluorescence imaging and surgical guidance. By this, it was possible to identify sentinel lymph nodes and to discriminate between metastatic tumor infiltration and inflammatory processes during surgery. This example of presurgical whole-body PET imaging together with subsequent intraoperative optical image-guided surgery in a larger animal shows the very high clinical potential of this approach.

In order to overcome the short circulation half-life and rapid RES accumulation of nanoparticles, resulting in a very low interaction probability of the imaging agent with the tissue to be visualized, the use of smaller particles (<12 nm) has been proposed [71, 73]. Furthermore, PEGs can be attached to the particle surface. This modification slows the particle resorption by liver and spleen [72, 73] but can in return result in a higher bone marrow uptake of the compounds* in vivo* [71, 73]. Particles comprising no targeting vector showing a rapid accumulation in the RES can however also be useful, especially for sentinel lymph node (SLN) mapping (Figure 1) [27, 60].

## 3. Conclusion

So far, the obtained results for hybrid PET/OI agents are variable. Nevertheless, some examples already show the high potential of these substances for target visualization with both imaging modalities. Future developments of dually labeled hybrid imaging agents for PET/OI exhibiting a favorable* in vivo* biodistribution and being applicable in a multimodal clinical setting face the challenge to introduce a radiolabel as well as a fluorescent reporter probe by at the same time preserving the favorable pharmacokinetic properties enabling a successful and specific target visualization.

Promising probes developed so far comprise dually labeled nanoparticles, antibodies, and peptides although the agents based on each of these substance classes require a careful optimization regarding the overall biodistribution of the hybrid agents. Thus, one focus of future developments could be the design of small molecules comprising the radionuclide as well as the NIR dye enabling the ideally site-specific, one-step dual-labeling of biomolecules, exerting only a minor structural alteration to the targeting vector and thus only a minor effect on its bioactivity resulting in highly potent compounds for hybrid imaging. In case of nanoparticles, the pharmacokinetic properties need to be optimized in order to minimize their uptake by the RES and to maximize their target-specific accumulation. Such developments could then result in hybrid imaging agents having a significant impact on whole-body* in vivo* target detection by PET as well as subsequent optical imaging-guided intraoperative curative surgery.
